# Data on chemical activation of Wnt/β-catenin during axolotl limb regeneration

**DOI:** 10.1016/j.dib.2017.02.048

**Published:** 2017-03-03

**Authors:** Sabina Wischin, Cristina Castañeda-Patlán, Martha Robles-Flores, Jesús Chimal-Monroy

**Affiliations:** a Departamento de Medicina Genómica y Toxicología Ambiental, Instituto de Investigaciones Biomédicas, Universidad Nacional Autónoma de México, Mexico DF 04510, Mexico; b Departamento de Bioquímica, Facultad de Medicina, Universidad Nacional Autónoma de México, Ciudad Universitaria,, Mexico DF 04510, Mexico

**Keywords:** Limb regeneration, Wnt signaling

## Abstract

Limb amputation in axolotls was performed to obtain data demonstrating that a chemical agonist of Wnt (int-related protein)/β-catenin signalling can have a role in axolotl limb regeneration (Wischin et al., 2017) [1]. The data revealed that active β-catenin protein was present during limb regeneration in some Leydig cells in the epithelium; after the chemical treatment, it was observed in more Leydig cells. In addition, the chemical agonist of Wnt generated distinct limb malformation.

**Table:** 

**Specification Table**	

Subject area	*Biology*
More specific area	*Regeneration*
Type of data	*Graphs, microscopy images*
How data was acquired	*Fluoview FV1000 laser confocal system (Olympus)*
Data format	*Processed*
Experimental factors	*Limb regeneration was studied in axolotls, the effect of Wnt agonist on limb regeneration was analysed*
Experimental features	*Immunofluorescence microscope, luciferase assay, RT-PCR, skeletal staining*
Data source location	*Instituto de Investigaciones Biomédicas, Universidad Nacional Autónoma de México*
Data accessibility	*The data are available with this article*

**Value of Data**

• The data showed that Wnt/β-catenin signalling was active throughout the limb regeneration process.
• It showed that the Wnt agonist activates Wnt/β-catenin signalling.
• This data can be used to determine that Wnt agonist has effects during axolotl limb regeneration.
• It can be used to determine that Wnt agonist is able to activate β-catenin signalling in Leydig cells.
• This data can be used to determine that Wnt agonist is able to induce limb malformations during limb regeneration.

## 1 Data

**Fig. 1:**
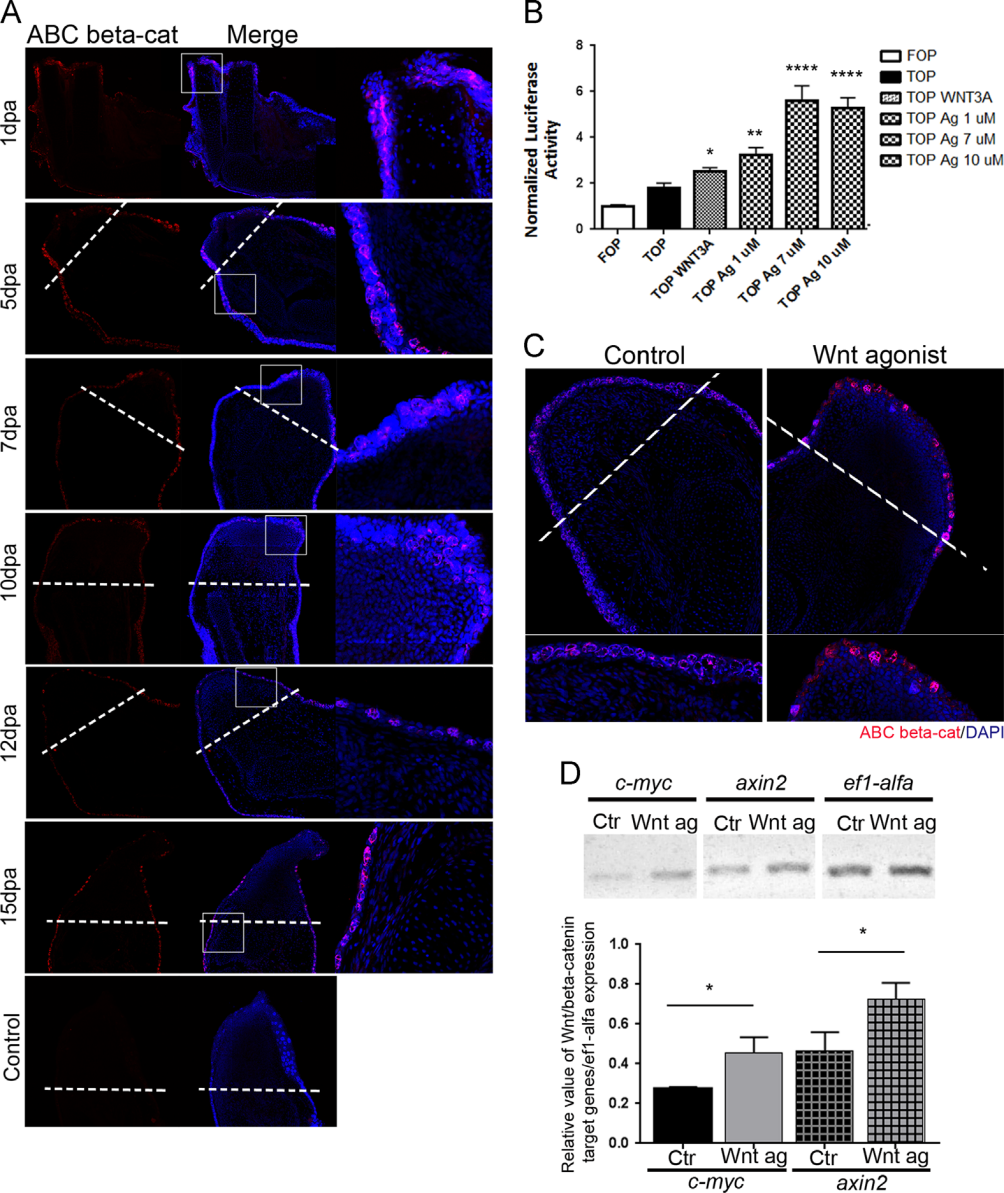
Active-β-catenin is present in limb epithelium Leydig cells and Wnt/β-catenin signalling is activated by Wnt agonist in the skin of regenerating limbs. (A) Histological sections of amputated limbs from 1, 5, 7, 10, 12 and 15 days post-amputation (dpa), showing the presence of active-β-catenin in Leydig cells nuclei of whole limb epithelized wounds. The dashed white line indicates the amputation plane. At least three limbs were taken for each time point. (B) Wnt agonist increased β-catenin-mediated transcriptional activity. RKO cells were cotransfected with a TCF reporter (TOPFlash) or flash reporter or Renilla luciferase (pRL) plasmids. At 24 h post-transfection, RKO cells were serum starved (2%) for 12 h; then, Wnt/β-catenin signalling was stimulated with WNT 3 A (100 ng/ml) or the Wnt agonist for an 8 h period. After incubation, cells were washed and lysed, and luciferase activity was assayed. The activity was normalized with respect to the activity of the Renilla luciferase. For TOP, the values were: 1.818±0.2015, for WNT 3 A: 2.514±0.1663; for Wnt agonist: 1 µM: 3.251±0.3122; for Wnt agonist: 7 µM: 5.615±0.6369, and for Wnt agonist: 10 µM: 5.265±0.4617. Results represent the means±SEM of at least four independent experiments *p<0.05, **p<0.01, ****p<0.0001. (C) Histological sections of amputated limbs from 12 dpa treated for 24 h with a 1 µM Wnt agonist. Chemical activation of Wnt/β-catenin signalling promotes the activation of β-catenin in Leydig cell nuclei as compared with controls. The dashed white line indicates the amputation plane (n=3/3). (D) RT-PCR analysis and its corresponding graph from 4 samples, showing that the chemical activation of Wnt/β-catenin signalling promotes *c-myc* and *axin2* expression in 12 dpa limb skin after a 12 h treatment. *ef1-α* was used as a control. The graph represents the relative value of *c-myc/ef1-α* and *axin2/ ef1-α* expression in the control and Wnt agonist-treated limb skins. The *c-myc* and *axin2* relative expression values for control limbs were 0.273±0.006 and 0.466±0.053, respectively. The relative expression values in Wnt agonist-treated limb skins were 0.456±0.045 for *c-myc* and 0.726±0.047 for *axin2*. *p<0.05 for both genes, n=3.

Data in Fig. 1A show that during the limb regeneration process, Wnt/β-catenin signalling was active in the epithelium (Fig. 1A). Active-β-catenin was observed in the nuclei of cells that apparently corresponded to Leydig gland cells but it was not observed in nuclei of other tissues (Fig. 1A).

Data in Fig. 1B showed that the Wnt agonist [2] could activate the Wnt/β-catenin signalling by TCF/LEF luciferase reporter (Fig. 1B).

**Fig. 2:**
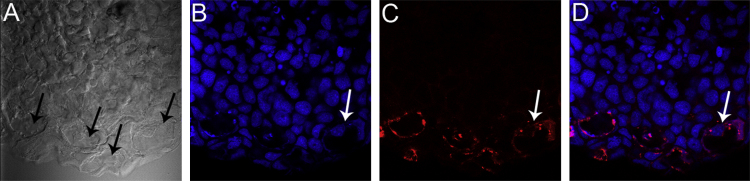
Active-β-catenin is present in limb Leydig cells. Epidermis of the regenerating axolotl skin. (A) Confocal bright field showing big round Leydig cells (white arrow). (B) DAPI. Arrow shows Leydig cell nuclei. (C) Active-β-catenin immunofluorescence. Arrow shows a positive mark in a Leydig cell. (D) Merge of B and C. Arrow shows the overlap of the nucleus with the positive mark of the anti β-catenin antibody in a Leydig cell.

Data in Figs. 1C and 2 show that the Wnt agonist (1 µM) treatment of amputated axolotls at 12 days post amputation (dpa) for 24 h activated β-catenin in Leydig cells nuclei, compared with controls, but not in mesenchymal cells of the blastema. Also, Wnt agonist increased expression of the target genes in the limb epithelium (Fig. 1D).

**Fig. 3:**
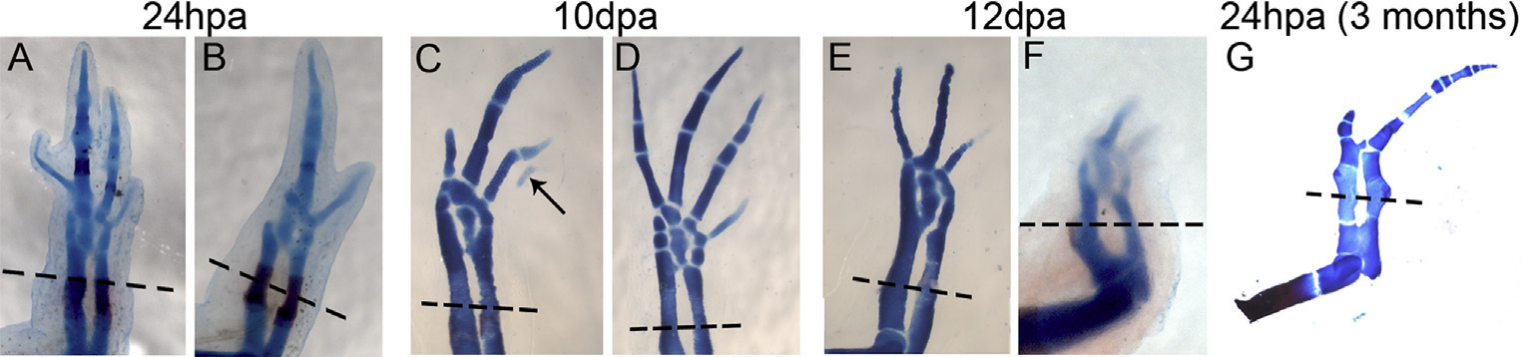
Limb malformations in regenerated limbs using the Wnt agonist treatment. Alcian blue/Alizarin red skeletal staining showing limb malformations obtained when the animals were treated at 24 hpa (A,B), 10 dpa (C,D) and 12 dpa (E,F). The anterior part of the limb is shown at the right and the posterior at the left. The arrow in C shows a slight Alcian blue stain. (G) Skeletal staining of a limb after three months after amputation, the axolotl was treated with the Wnt agonist for three days at 24 hpa.

Data in Fig. 3A show that Wnt agonist caused limb malformations. In animals treated 24-hours post amputation for 24 h, the anterior-most skeletal elements of the regenerated limb were missing (Fig. 3A and B). Animals treated at 10 dpa for 24 h developed three digits and one more, which could not be clearly identified as a digit (Fig. 3C) or did not develop completely (Fig. 3D). Limb of animals treated at 12 dpa presented only three slender digits with no joints (Fig. 3E) or two small digits with no joints (Fig. 3F). Data in Fig. 3G show that only one individual could regenerate after three months after amputation, however, it only developed one digit and no carpus or ossification was observed (Fig. 3G).

## 2 Experimental design, materials and methods

### 2.1 Animal maintenance and treatment

All experiments were performed on 5–6.5 cm axolotls (*Ambystoma mexicanum*). One forelimb was amputated at mid-zeugopod after they were anesthetised with 0.05% Tricaine (Sigma-Aldrich, St. Louis, MO, USA). The animals were placed in the solution with the Wnt agonist (Calbiochem, Billerica, MA, USA) on different days after amputation. The vehicle, dimethyl sulfoxide (DMSO) was used as control. Limbs were amputated at the base of the arm and collected at the end of the experiment.

This research protocol was reviewed and approved by the Institutional Review Board for the Care and Use of Laboratory Animals of the Instituto de Investigaciones Biomédicas, UNAM. All experiments in the present study were carried out in accordance with the approved guidelines.

### 2.2 Tissue staining and skeletal preparation

Tissue samples were fixed in 4% paraformaldehyde at 4 °C overnight and processed to be embedded in Paraplast (Sigma-Aldrich) and sectioned into 7 µm for tissue staining. They were stained with the Masson Trichrome technique. For skeleton staining, limbs were fixed in 95% ethanol and permeabilized overnight with acetone. Later, collected limbs were stained in an Alcian blue/Alizarin red solution for three days and were cleared in 1% KOH/20% glycerol and stored in 50% ethanol/50% glycerol.

### 2.3 Immunohistochemistry

Active-β-catenin (1:200; 05–665, Millipore, Billerica, MA) and 1:500 anti-rabbit Alexa 488 (A21206, Invitrogen, Carlsbad, CA, USA) was evaluated. Fluorescent immunostainings were observed under laser confocal system (Fluoview FV1000, Olympus). Anti-β-catenin immunohistochemistry was performed in cryosections of 40 µm.

### 2.4 RT-PCR and luciferase assay

RNA expression detected RT-PCR using RNA collected from eight 12-day post-amputated axolotl limb skins, from animals treated either with the Wnt agonist or with DMSO. We used the following primers:

*c-myc* forward primer 5′-TGACCCTTCAGTGGTCTTCC-3′
*c-myc* reverse primer 5′-CGCCTCTTGTCGTTCTCTTC-3′
*axin2* forward primer 5′-GAGTCTGACGCTTGGACACT-3′
*axin2* reverse primer 5′-AGAAACTCGGTGAGTGGCATT-3′

The *ef1*α primers and PCR conditions were obtained from [3].

A luciferase assay and RKO (colon cancer cell line) cell culture were performed to determine the efficiency of the drug to activate the Wnt/β-catenin signalling as described previously [4].
