# Supplementary material for: Data on chemical activation of Wnt/β-catenin during axolotl limb regeneration
Source: Data Brief. 2017 Mar 3;11:562–6. doi: 10.1016/j.dib.2017.02.048 (PMC5357679; doi:10.1016/j.dib.2017.02.048)
Supplement: Supplementary file 1 — Supplementary material [file mmc1.docx]

Mexico City, January 26, 2017

Marina Ostankovitch, PhD

Senior Scientific Editor

Data in Brief

Dear Dr. Ostankovitch,

The authors of manuscript DIB-D-16-00903R1 have no conflict of interest to declare.

Sincerely

Jesús Chimal-Monroy

Instituto de Investigaciones Biomédicas, UNAM

jchimal@unam.mx
